# Diabetes in young adult men: social and health-related correlates

**DOI:** 10.1186/s12889-016-3704-7

**Published:** 2016-10-31

**Authors:** Rachel L. Koelmeyer, Shyamali C. Dharmage, Dallas R. English

**Affiliations:** 1 Centre for Epidemiology and Biostatistics, The University of Melbourne, Melbourne, VIC 3010 Australia; 2 School of Clinical Sciences at Monash Health, Monash University, Clayton, 3168 VIC Australia; 3 Murdoch Childrens Research Institute, Parkville, 3052 VIC Australia; 4 Cancer Epidemiology Centre, Cancer Council Victoria, Melbourne, 3004 VIC Australia

## Abstract

**Background:**

Diabetes is a global public health issue. It is associated with significant disability, morbidity and mortality risks and substantial healthcare costs. Of great concern is the fact that its prevalence is rising, particularly amongst the young, while epidemiological data regarding the incidence, prevalence and complications of early-onset type 2 diabetes is noted to be sparse.

**Methods:**

We used data from the baseline wave of Ten to Men, a national cohort study of Australian males, to investigate the social and health-related correlates of Australian males aged 18–49 years reporting being diagnosed with diabetes.

**Results:**

The estimated prevalence of a self-reported diabetes diagnosis amongst Australian males aged 18–49 years was 2.95 % (95 % CI: 2.54–3.43 %). Within this age group, approximately 75 % of those diagnosed with diabetes are expected to be living with a known diagnosis of type 2 diabetes; the remainder are expected to be living with type 1 diabetes. Of the 20 social and health-related factors considered, we found evidence to support the association of eighteen factors after adjusting for age and body mass index. The strongest correlates of reporting a diabetes diagnosis, associated with a ≥2-fold increase in the odds of reporting diabetes were being aged 35–49 years, being unemployed, being obese, seeing a doctor for a check-up more frequently, reporting comorbid high blood pressure or physical or mental health comorbidities and worse self-rated and physical health status.

**Conclusion:**

Australian males aged 18–49 years who are living with a known diagnosis of diabetes are more likely to be socio-economically disadvantaged and suffer substantially worse health status than Australian males aged 18–49 years living without a diabetes diagnosis. Based on the associations detected in this study, older, single males living in regional areas who are socioeconomically disadvantaged, obese and/or who have other comorbidities may be an important subgroup to target for diabetes screening, disease management and prevention efforts.

## Background

Diabetes, a chronic condition characterised by high blood glucose levels [1] and associated with a number of serious sequelae such as cardiovascular comorbidities, peripheral neuropathy, retinopathy and kidney disease [2, 3], is a global public health concern [4]. In 2015, it was estimated that 415 million people globally were living with diabetes and 5 million deaths were attributable to diabetes [5]. Additionally, health expenditure due to diabetes was estimated at US$673 million in 2015 [5]. Onset of type 2 diabetes, the predominant form of diabetes, occurs most commonly in adulthood and is characterised by reduced insulin production and/or reduced insulin responsiveness [1]; in contrast, type 1 diabetes, accounting for approximately 5 – 10 % of all diabetes cases [6], is characterised by autoimmune destruction of insulin-producing cells and onset occurs most commonly in childhood [1]. Type 2 diabetes is potentially preventable given the role of lifestyle factors such as obesity and lack of physical activity in its pathogenesis [1].

Internationally, the prevalence of type 2 diabetes is rising, particularly in children, adolescents and younger adults [5–8]. Early-onset type 2 diabetes has similar pathophysiology to later-onset diabetes, with both genetic and lifestyle risk factors being implicated [3, 9, 10]. However, recent evidence suggests that early-onset diabetes is more aggressive, associated with more rapid decline in beta-cell function, greater likelihood of insulin therapy and greater risk of comorbidities and death [10–13]. Epidemiological data regarding the incidence and prevalence of early-onset type 2 diabetes and its associated complications is sparse [3, 10]. While some international studies have investigated early-onset diabetes [11, 14–17], as far as we are aware, early-onset diabetes has only been specifically investigated in Australia in an Indigenous population [18]. The prevalence of type 2 diabetes is higher in men compared with women [6, 19], making males a priority population for disease prevention efforts.

In this paper, we use data from the baseline wave of Ten to Men, a national longitudinal study on male health, to investigate the social and health-related correlates of self-reported diabetes in younger adult males, with a view to identifying a subgroup of Australian males and priority risk factors to target for disease prevention efforts and optimal management of diabetes in young men.

## Methods

### Ten to men

The design, setting and characteristics of participants of the Ten to Men study are reported elsewhere in this issue [20]. In brief, Australian males aged 10–55 years were recruited in 2013 and 2014 via household recruitment from the Australian Statistical Geography Standard (ASGS) [21] major city, inner regional and outer regional areas of Australia. The sampling plan was designed to oversample males living in inner and outer regional areas. The final sample was drawn from 622 SA1s (a sampling unit defined within the ASGS [21]), including 363 major city SA1s, 144 inner regional SA1s and 115 outer regional SA1s. A total of 104,484 households were approached, from which 15,988 Australian males were recruited, resulting in a response fraction of 35 % by confirmed in-scope males.

### The current analysis

This analysis is based on men aged 18 to 49 years at baseline. All participants answered a self-completed questionnaire about their physical and mental health status, health-related behaviours, family and social life and health service use. Copies of the questionnaires are available online [22].

## Variables

### Outcome variable

Two questions were asked about diabetes: “Has a doctor or other health professional ever told you that you had this condition?” and “Have you been treated for or had symptoms of this condition in the past 12 months?” For the purpose of this study, diabetes was defined as reporting ever being diagnosed with diabetes by a doctor or health professional. The questions did not differentiate type 1 and type 2 diabetes; however, based on data from the Australian Health Survey, Australia’s largest national health survey [23], it is anticipated that approximately one-quarter of respondents in the 18–49 year age group would have been diagnosed with type 1 diabetes and approximately three-quarters would have been diagnosed with type 2 diabetes [24].

### Potential correlates

Sociodemographic variables included age, country of birth, parents’ country of birth, region of residence, marital status, highest education level, employment status, before-tax household income and number of life events experienced in the past 12 months. Region of residence was classified in accordance with the ASGS [21].

Health-related behaviour measures included smoking status, fruit and vegetable consumption, level of physical activity in the last week (measured using the Active Australia Survey [25]), body mass index (BMI; derived from self-reported height and weight and classified into healthy weight range or below, BMI ≤25 kg/m^2^; overweight, BMI 25 - <30 kg/m^2^; obese, BMI ≥30 kg/m^2^ or missing), and frequency of visiting a family doctor for a check-up.

Health and wellbeing status variables included self-rated health, the physical component score (PCS) and mental component score (MCS) measures from the SF-12 [26], self-reported lifetime diagnosis of potential comorbidities (angina, anxiety disorder, arthritis, asthma, chronic bronchitis, chronic obstructive pulmonary disease, cancer, cataract, depression, eczema, high blood pressure, high cholesterol, heart attack, heart failure, schizophrenia, sleep apnoea and stroke) and subjective wellbeing, as measured using the Personal Wellbeing Index for Adults (English PWI-A, 4^th^ edition; PWI SWS) [27, 28].

### Statistical methods

Descriptive statistics were used to report the proportion of Australian males with particular social and health-related characteristics who reported a diabetes diagnosis. The association of the social and health-related variables with reporting diabetes were assessed using logistic regression, with and without adjustment for key type 2 diabetes risk factors (age: age </≥ 35 years; BMI: non-obese, obese and missing). To assess differences in the prevalence of diabetes based on comorbidity status, the proportion of respondents with and without a lifetime diagnosis of each comorbidity who reported diabetes was calculated using survey design commands to adjust for the survey design and apply population weights and then directly standardised to the age distribution of the 2011 Census population of Australian males aged 18–49 years [29] and using the age groups 18–24 years, 25–34 years, 35–44 years and 45–49 years. *χ* ^2^ tests were used to assess the statistical evidence of a difference in the proportions. Missing data were excluded from the analysis except when adjusting for BMI. Most variables had a low level of missing data, with an average proportion of 2.8 % missing data per variable. Four variables had >5 % missing data: highest education level (6.5 %), level of physical activity in the last week (10.4 %), BMI (12.7 %) and before-tax household income (15.8 %). All analyses were conducted using StataSE 13.1 (Stata Corp LP, College Station, TX, USA).

## Results

### Prevalence of diabetes in younger Australian males

There were 11,307 males aged 18–49 years, of whom 11,075 provided data on whether or not they had ever been diagnosed with diabetes (97.9 %). A history of diabetes was reported by 334 males, of whom 85.6 % reported treatment for or symptoms of diabetes in the 12 months prior to baseline. After taking account of the survey design and using population weights, this equated to an estimated population prevalence of ever being diagnosed with diabetes of 2.95 % (95 % CI: 2.54 – 3.43 %).

### Social correlates of reporting diabetes

Table 1 provides an overview of the social correlates of reporting diabetes, before and after adjusting for age and BMI. Even after adjustment for age and BMI, there was moderate to strong statistical evidence of associations between all social factors other than education and increased odds of reporting diabetes. Particular social characteristics found to be associated with increased odds of reporting diabetes included being aged ≥35 years, being born in or having parents who were born in a country associated with increased risk of diabetes, living in a regional area, being unemployed, reporting a weekly before-tax income of < AU$100,000 and having had a higher than average number of life events. The strongest correlates of reporting diabetes, associated with a ≥2-fold difference in the odds of reporting diabetes were being aged 35–49 years and reporting being unemployed.

**Table 1:** Social correlates of reporting diabetes (*n* = 11,075)

Potential correlate	Proportion Reporting Diabetes (%)	Association with Reporting Diabetes^a^ Odds Ratio (95 % Confidence Interval; *p* value)	
		Unadjusted	Adjusted^b^
Known risk factors			
Age			
18 – 24 years, *n* = 1,951	1.44	1.0 (Not Applicable)	1.0 (Not Applicable)
25 – 34 years, *n* = 3,035	1.55	1.08 (0.67 – 1.73; 0.748)	1.0 (0.62 – 1.60; 0.991)
35 – 44 years, *n* = 4,053	3.45	2.46 (1.63 – 3.70; <0.001)	2.16 (1.42 – 3.27; <0.001)
45 – 49 years, *n* = 2,036	5.84	4.3 (2.81 – 6.47; <0.001)	3.61 (2.36 – 5.51; <0.001)
County of birth			
At risk region^c^, *n* = 1,245	3.94	1.37 (1.0 – 1.87; 0.045)	1.75 (1.28 – 2.41; 0.001)
Other country, *n* = 9,823	2.90	1.0 (Not Applicable)	1.0 (Not Applicable)
Parents’ country of birth			
Not from at risk region^c^, *n* = 9,073	2.90	1.0 (Not Applicable)	1.0 (Not Applicable)
One from at risk region^c^, *n* = 340	3.24	1.12 (0.61 – 2.07; 0.717)	1.25 (0.67 – 2.34; 0.474)
Both from at risk region^c^, *n* =1,518	3.62	1.26 (0.94 – 1.69; 0.127)	1.54 (1.14 – 2.08; 0.005)
Other Potential Correlates			
ASGS region of residence			
Major city, *n* =6,503	2.77	1.0 (Not Applicable)	1.0 (Not Applicable)
Inner regional, *n* = 2,510	3.82	1.40 (1.09 – 1.80; 0.009)	1.29 (1.0 – 1.67; 0.048)
Outer regional, *n* =2,053	2.83	1.02 (0.76 – 1.38; 0.891)	0.90 (0.67 – 1.22; 0.503)
Marital status			
Married/de facto rel., *n* = 7,136	3.11	1.0 (Not Applicable)	1.0 (Not Applicable)
Previously married, *n* = 555	4.86	1.59 (1.06 – 2.40; 0.026)	1.40 (0.92 – 2.11; 0.114)
Never married, *n* = 3,308	2.51	0.80 (0.62 – 1.03; 0.090)	1.44 (1.08 – 1.92; 0.013)
Highest education level			
Bachelor degree or higher, *n* =2,788	2.73	1.0 (Not Applicable)	1.0 (Not Applicable)
Non-degree qualification, *n* = 4,836	3.02	1.11 (0.84 – 1.47; 0.464)	0.92 (0.69 – 1.23; 0.575)
Secondary school or less, *n* = 2,766	3.25	1.20 (0.88 – 1.64; 0.249)	1.13 (0.82 – 1.56; 0.449)
Employment status			
Employed, *n* = 9,292	2.49	1.0 (Not Applicable)	1.0 (Not Applicable)
Unemp. (looking for work), *n* = 962	5.30	2.20 (1.61 – 3.0; <0.001)	2.75 (1.99 – 3.80; <0.001)
Unemp. (not looking), *n* =589	6.96	2.93 (2.08 – 4.14; <0.001)	2.94 (2.07 – 4.20; <0.001)
Before-Tax Income			
AU$100 K+, *n* = 4,422	2.40	1.0 (Not Applicable)	1.0 (Not Applicable)
AU$60 K - < AU$100 K, *n* = 2,637	3.19	1.34 (1.0 – 1.79; 0.048)	1.39 (1.03 – 1.86; 0.029)
< AU$60 K, *n* = 2,291	3.97	1.68 (1.27 – 2.24; <0.001)	1.93 (1.44 – 2.59; <0.001)
Above-average number of life events, *n* = 163	3.18	1.14 (0.92 – 1.43; 0.236)	1.28 (1.02 – 1.60; 0.035)

^a^Where not stated, the reference category is the absence of the characteristic

^b^After adjusting for age and body mass index

^c^Countries/regions associated with increased risk of diabetes: Asia, Middle East, North Africa or Southern Europe

### Health-related correlates of reporting a diabetes diagnosis

Table 2 provides an overview of the health-related correlates of reporting a diabetes diagnosis, before and after adjusting for age and BMI. All factors considered, other than daily fruit and vegetable consumption, were found to be associated with increased or decreased odds of reporting diabetes, after adjusting for age and BMI. Reporting current smoking, a sedentary lifestyle, obesity, ever being diagnosed with high blood pressure, an above-average number of comorbidities and more frequent check-ups with a doctor were associated with increased odds of reporting diabetes. In contrast, males aged 18–49 years who were in better health (reported good to excellent health, an average or above physical or mental component score and/or an average or above subjective wellbeing score) were substantially less likely to report diabetes, indicating that younger Australian males living with diabetes had significantly worse health and wellbeing status. The strongest health-related correlates of reporting diabetes, associated with a ≥2-fold change in the odds of reporting diabetes were reporting being obese, having other comorbidities, physical health status and frequency of check-ups with their doctor.

**Table 2:** Health-related correlates of reporting diabetes (*n* = 11,075)

Potential correlate	Proportion Reporting Diabetes (%)	Association with Reporting Diabetes^a^ Odds Ratio (95 % Confidence Interval; *p* value)	
		Unadjusted	Adjusted^b^
Known Risk Factors			
Smoking status			
Never smoked, *n* = 6,320	2.52	1.0 (Not Applicable)	1.0 (Not Applicable)
Ex-smoker, *n* = 2,415	3.81	1.53 (1.18 – 1.99; 0.001)	1.17 (0.90 – 1.53; 0.251)
Current smoker, *n* = 2,175	3.72	1.50 (1.14 – 1.97; 0.004)	1.38 (1.04 – 1.81; 0.024)
Less than daily fruit/veg. consumption, n = 415	3.86	1.30 (0.78 – 2.17; 0.316)	1.31 (0.78 – 2.21; 0.306)
Physical activity level			
Sufficient for health, *n* = 5,663	2.38	1.0 (Not Applicable)	1.0 (Not Applicable)
Insufficient for health, *n* = 2,917	2.91	1.23 (0.93 – 1.62; 0.142)	1.03 (0.78 – 1.36; 0.825)
Sedentary, *n* = 1,381	5.14	2.22 (1.65 – 2.98; <0.001)	1.66 (1.23 – 2.25; 0.001)
BMI			
Healthy range or less, *n* = 3,340	1.53	1.0 (Not Applicable)	1.0 (Not Applicable)
Overweight, *n* = 4,116	2.02	1.33 (0.93 – 1.89; 0.115)	1.12 (0.78 – 1.59; 0.537)
Obese, *n* = 2,235	6.76	4.67 (3.39 – 6.45; <0.001)	3.83 (2.77 – 5.31; <0.001)
Missing, *n* = 1,384	3.54	2.37 (1.59 – 3.52; <0.001)	2.35 (1.58 – 3.50; <0.001)
High blood pressure (ever), *n* = 1,468	10.22	6.30 (5.02 – 7.90; <0.001)	4.38 (3.44 – 5.56; <0.001)
Other Potential Correlates			
Reported good to excellent self-rated health, *n* = 10,153	2.22	0.17 (0.13 – 0.21; <0.001)	0.23 (0.18 – 0.29; <0.001)
Average or above SF-12 PCS, *n* = 6,640	1.54	0.27 (0.22 – 0.35; <0.001)	0.35 (0.27 – 0.45; <0.001)
Average or above SF-12 MCS, *n* = 6,320	2.37	0.60 (0.48 – 0.76; <0.001)	0.62 (0.50 – 0.78; <0.001)
Average or above PWI SWS, *n* = 6,378	2.09	0.46 (0.36 – 0.57; <0.001)	0.51 (0.41 – 0.64; <0.001)
Above-average number of comorbidities, *n* = 3,545	6.01	4.19 (3.32 – 5.28; <0.001)	3.28 (2.59 – 4.15; <0.001)
Check-up with doctor			
Never, *n* = 4,019	0.90	1.0 (Not Applicable)	1.0 (Not Applicable)
Less than yearly, *n* = 2,925	1.68	1.89 (1.22 – 2.91; 0.004)	1.75 (1.13 – 2.70; 0.012)
Yearly, *n* = 2,175	2.90	3.30 (2.18 – 4.99; <0.001)	2.82 (1.86 – 4.27; <0.001)
More than once a year, *n* = 1,528	11.26	14.0 (9.75 – 20.2; <0.001)	11.0 (7.63 – 15.9; <0.001)

^a^Where not stated, the reference category is the absence of the characteristic

^b^After adjusting for age and body mass index

### Proportion reporting diabetes by comorbidity status

Table 3 provides details of the proportion of males reporting diabetes based on their comorbidity status. Of the potential comorbidities, there was strong statistical evidence that males who reported being diagnosed with all but two other comorbidities assessed (asthma and eczema), were more likely to report diabetes, after adjusting for age using direct standardisation. This included both physical and mental health comorbidities.

**Table 3:** Differences in proportion reporting diabetes by comorbidity status (*n* = 11,075)

Potential comorbidity	Potential comorbidity status				Evidence of difference in proportion with diabetes *P* value from *χ*2 test
	Never diagnosed		Lifetime diagnosis		
	Number of respondents	% with early-onset diabetes^a^	Number of respondents	% with early-onset diabetes^a^	
Angina	10,941	2.5	98	17.5	<0.001
Anxiety disorder	9,595	2.3	1,440	4.1	<0.001
Arthritis	10,160	2.4	876	5.2	<0.001
Asthma	8,487	2.5	2,548	3.0	0.215
Cancer	10,806	2.4	231	12.3	<0.001
Cataract	10,960	2.5	86	15.1	<0.001
Chronic bronchitis	10,537	2.4	486	5.1	<0.001
Chronic Obstructive Pulmonary Disease	10,993	2.5	50	18.2	<0.001
Depression	8,977	2.0	2,070	5.2	<0.001
Eczema	9,825	2.5	1,207	3.0	0.320
Heart attack	10,972	2.4	81	28.7	<0.001
Heart failure	10,998	2.5	49	35.5	<0.001
High blood pressure	9,579	1.7	1,468	8.7	<0.001
High cholesterol	9,608	1.7	1,435	7.7	<0.001
Schizophrenia	10,952	2.4	84	16.9	<0.001
Sleep apnoea	10,199	2.2	841	8.2	<0.001
Stroke	10,984	1.9	60	19.0	<0.001

^a^Proportion adjusted for survey design characteristics and then standardised based on the age distribution of Australian males aged 18- < 50 years based on the 2011 Census

## Discussion

Approximately one in 33 men aged 18–49 years were living with a known diagnosis of diabetes, equating to an estimated population prevalence of 2.95 % in Australian men aged 18–49 years. Of the twenty social and health-related factors considered, we found evidence that eighteen were associated with a known diagnosis of diabetes. The strongest correlates of diabetes, associated with a ≥2-fold increase in the odds of diabetes were being aged 35–49 years, being unemployed, being obese, seeing a doctor for a check-up more frequently, reporting comorbid high blood pressure or physical or mental health comorbidities and worse self-rated and physical health status.

Particularly notable findings are the inequities that younger adult males living with diabetes appear to face in terms of their socioeconomic circumstances (more likely to be unemployed and have a lower income) and health status (significantly worse physical and mental health status with greater likelihood of reporting most other comorbidities after adjusting for age distribution), despite still being relatively young. Based on this study, younger adult males living with diabetes are likely to experience a number of comorbidities, both physical and mental, and are therefore likely to have complex healthcare needs. Our findings are generally consistent with other studies in terms of known risk factors for type 2 diabetes [3, 30], increased prevalence of a number of comorbidities commonly associated with diabetes [5, 10, 11] and the health system burden associated with diabetes [3, 5]. This includes the association of worse socioeconomic status [31], cardiovascular comorbidities [11] and worse physical and mental health with a known diabetes diagnosis [32]. Our study is unusual in terms of exploring the association of diabetes with a more general set of comorbidities and health status measures and exploring socioeconomic correlates in greater depth.

This study has some limitations. Our analyses were based on self-report data, such that some respondents may have been misclassified as living without diabetes or another comorbidity since they may experience the condition without a formal diagnosis. Furthermore, a self-reported diagnosis may not always represent clinically-important disease (for example, a patient experiencing symptoms of depression without meeting clinical thresholds for depression). Additionally, since the study questionnaire did not distinguish type 1 diabetes from type 2 diabetes and given the expected ratio of type 1:type 2 diabetes cases of 1:3, even though our study focused on factors that are associated with type 2 diabetes [3, 5, 30]; it is likely that the strength of the association of such factors with type 2 diabetes has been under-estimated in this study.

A further limitation is that since this study is based on cross-sectional and lifetime frequency data, we cannot be certain which of the associated factors are causes of diabetes, sequelae of diabetes or concurrent experiences. However, based on the findings of our study, assessing how diabetes impacts on socioeconomic status and whether a reported diagnosis of diabetes predisposes younger adult men to other comorbidities beyond commonly-associated conditions such as cardiovascular comorbidities warrants further investigation using longitudinal data.

## Conclusions

Australian males aged 18–49 years and living with a diabetes diagnosis are more likely to be socio-economically disadvantaged and suffer worse health status than Australian males aged 18–49 years who are living without a diabetes diagnosis. Based on the associations detected in this study, single males living in regional areas who are socioeconomically disadvantaged and/or inactive or obese or who have other comorbidities may be an important subgroup to target for diabetes screening, disease management and prevention efforts.

## Abbreviations

ASGS: Australian Statistical Geography Standard
BMI: Body mass index
MCS: SF-12 mental component score
PCS: SF-12 physical component score
PWI SWS: Personal wellbeing index subjective wellbeing score
PWI-A: Personal wellbeing index for adults
